# New single nucleotide variation in the promoter region of androgen receptor (AR) gene in hypospadic patients

**Published:** 2014-03

**Authors:** Nasim Borhani, Marefat Ghaffari Novin, Mehdi Manoochehri, Mohsen Rouzrokh, Bahram Kazemi, Ameneh Koochaki, Ahmad Hosseini, Reza Masteri Farahani, Mir Davood Omrani

**Affiliations:** 1 *Cellular and Molecular Biology Research Center, Shahid Beheshti University of Medical Sciences, Tehran, Iran. *; 2 *Department of Cell Biology and Anatomy, School of Medicine Shahid Beheshti University of Medical Sciences, Tehran, Iran.*; 3 *Department of Pediatric Surgery, Mofid Children's Hospital, School of Medicine, Shahid Beheshti University of Medical Sciences, Tehran, Iran.*; 4 *Department of Genetics, School of Medicine, Shahid Beheshti University of Medical Sciences, Tehran, Iran.*

**Keywords:** *Hypospadias*, *Androgen receptor gene*, *Promoter*

## Abstract

**Background:** Hypospadias is one of the most common congenital abnormalities in the male which is characterized by altered development of urethra, foreskin and ventral surface of the penis. Androgen receptor gene plays a critical role in the development of the male genital system by mediating the androgens effects.

**Objective:** In present study, we looked for new variations in androgen receptor promoter and screened its exon 1 for five single nucleotide polymorphisms (SNP) in healthy and hypospadias Iranian men.

**Materials and Methods: **In our study, at first DNA was extracted from patients (n=100) and controls (n=100) blood samples. Desired fragments of promoter and exon 1 were amplified using polymerase chain reaction. The promoter region was sequenced for the new variation and exone 1 screened for five SNPs (rs139767835, rs78686797, rs62636528, rs62636529, rs145326748) using restriction fragment length polymorphism technique.

**Results: **The results showed a new single nucleotide variation (C→T) at -480 of two patients’ promoter region (2%). None of the mentioned SNPs were detected in patients and controls groups (0%).

**Conclusion:** This finding indicates that new single nucleotide polymorphism in androgen receptor promoter may have role in etiology of hypospadias and development of this anomaly.

This article extracted from Ph.D. thesis. (Nasim Borhani)

## Introduction

Hypospadias is the most common congenital malformation of the male external genitalia in which the urethral meatus opens on the ventral side of the penis (1). It affects about three to eight in 1000 newborn males (2). The location of the urethral meatus can differ from the penile glans of the penis to the scrotal or perineal region. The glandular and penile types are more common and considered as isolated hypospadias, whereas scrotal and prineal types are sporadic and considered as severe hypospadias (3). The androgen-androgen receptor interaction is critical for male external genitalia formation (4). 

Impaired signaling through the androgen receptor (AR) leads to failure of urethral fold formation and fusion and consequently leads to hypospadias (5). The *AR* gene is located on chromosome Xq11-12 and consists of eight exons, in which exon 1 encodes the transactivation domain that activates transcription of several different *AR*-responsive downstream genes (   6 , 7). The results of some previous studies showed the reduced androgen receptor expression level and androgen-binding capacity in genital skin fibroblasts of hypospadic patients (   8 , 9). These results suggest that mutations of the promoter region and transactivation domain of the *AR *gene could hinder or decrease the transcription machinery affinity for binding toward regulatory elements, thereby reducing the total amount of *AR* gene expression in the genital system. Therefore, in the present study, exon1was screened for five single nucleotide polymorphisms (SNP), and in addition, *AR* gene promoter region was scanned for new variations in hypospadias disease in Iranian population.

## Materials and methods

**Patients and controls**

The patients in this analytical association study refered to the Mofid’s children Hospital (the second referral center in Iran, Tehran) between March 2012 to August 2012. The patients should have criteria including diagnosis by urologist and having no sign of other genetic or congenital disease or genital malformations; (the properties distribution of patients in our study are listed in table I).

The controls were selected from healthy males in Shahid Beheshti School of Medicine. The control samples should not have any sign of genetic disease, genital malformations or any addiction. Peripheral blood samples of hypospadic patients (n*=*100) and control group (n*=*100) were taken using venoject tube containing EDTA (0.5 M). 

**DNA extraction and polymerase chain reaction (PCR)**

Genomic DNA was extracted from peripheral blood of patients and controls using the whole blood extraction kit (Dr. Gen TLE, Takara, Japan). Nucleotides -756 to -366 of the AR 5’ promoter region (based on Ensemble.org genome browser) were amplified using the primer pair: 5’TCTCCAAA GCCACTAGGCAG3’ (sense) and 5’ACCGAA GAGGAAAGGGCAGCTC3’ (antisense). Nucleotides +604 to +888 of the *AR* transactivation domain were amplified using the primer pair: 5’TCCAGAGCGTGCGCGAA GTG3’ (sense) and 5’CCGACTGCGGCTGT GAAGGT3’ (antisense The 30 µl reaction PCR reactions contained 100 ng genomic DNA, 100 pmol mixed primer pair, distilled water and master mix (Ampliqon, Denmark). 

**Restriction fragment length polymorphism (RFLP) analysis**

For transactivation domain genotyping, PCR, RFLP and 8% polyacrylamide gel electrophoresis (PAGE) were used. The RFLP reaction condition was detailed and presented in Table II.

**Promoter region sequencing**

For AR promoter region sequencing, PCR products were purified using DNA extraction kit (Fermentas, Lithuania) and then scanned for variations using direct sequencing (Applied Bio systems 3730/3730xl DNA Analyzers Sequencing, Bioneer, Korea).

**Ethical considerations**

The informed consent was obtained from all adult participants (control groups) and from the parents or legal guardians of minors (patients). This study had been reviewed and approved by a certified Ethical Committee in Shahid Beheshti University of Medical Sciences.

## Results

**RFLP results**

The 285 bp PCR product containing five SNP sites was digested using suitable enzymes; SsiI, PstI, CaiI, SfaNI, and BseG1. The two SNPs rs62636528, and rs62636529 produce gain of sites compared to wild type allele, while rs78686797, rs139767835, and rs145326748 supposed to have loss of sites in digestion with the mentioned enzymes (Table II). The SsiI, PstI, and BseGi could cut wild type alleles and produce 2-3 shorter fragments that could be visualized on PAGE; while the CaiI and SfaNI will cut mutant alleles and leave the intact wild types.

**Sequencing results**

**Figure 1:**
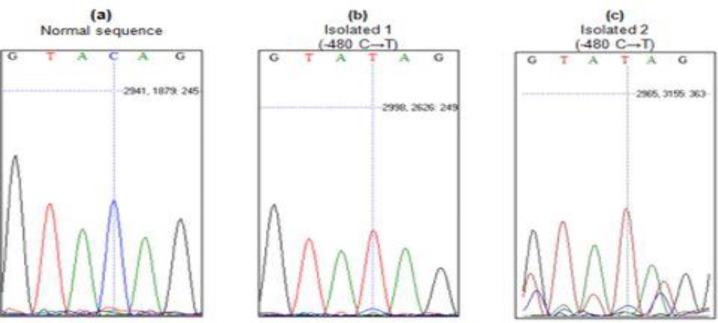
**(a)** Normal sequence from control group (C nucleotide in -480), **(b)** Isolated 1 from patient group (C→T in -480), **(c) **Isolated 2 from patients group (C→T in -480).

*AR* gene promoter region was scanned for new variations in subjected samples and a new single nucleotide polymorphism (SNP) was detected in two patient samples (2%) (One glandular and one distal penile type) at position -48^o^C→T (the normal sequence and two sequences curves with a new nucleotide variation are shown in Figure 1).

**Table I:** The properties distribution of patients. The different number of each hypospadiuas subtype, presence (yes) or absence (no) of Parental Consanguinity and Family history are depicted

**Type**	**Patients number** *	**Parental consanguinity**		**Family history**	
		**Yes**	**No**	**Yes**	**No**
Penoscrotal	13	1	12	1	12
Proximal penile	2	-	2	-	2
Mid shaft	7	2	5	-	7
Distal penile	53	8	45	5	48
(sub)Coronal	5	1	4	-	5
Glandular	20	2	18	1	19

* Total patients number = 100

**Table II:** Restriction fragment length polymorphism protocol for exon 1 genotyping

**SNP**	**Restriction enzyme**	**Gain of site**	**Loss of site**
rs139767835	SsiI a		●
rs78686797	PstI a		●
rs62636528	CaiI a	●	
rs62636529	SfaNI^b^	●	
rs145326748	BseG1 a		●

All reactions were prepared in order to the enzymes protocol.

a Fermentase (Ukraine)

b Biolabs (UK)

## Discussion

*AR* gene is a member of the nuclear receptor superfamily that is located on Xq11-12. This receptor plays a critical role in male sexual differentiation by mediating the biological effects of androgens (10). Early development of external genital system is similar for both sexes but urethral folds fusion and masculinization process is androgen-androgen receptor dependant phase. Therefore any changes in *AR* amount or structure may disrupt this patterning (11). In our knowledge, we scanned the promoter region of *AR* for the first time in hypospadic patients and we found a new single nucleotide polymorphism in two patients at -480 using direct sequencing. There have been many reports on AR promoter sequencing. 

In 2006, Waltering *et al* scanned *AR* gene promoter and untranslated region in 44 clinical prostate cancer specimens and 36 normal controls; but they did not find any different sequence variations (12). Likewise, Ghadessy *et al* screened promoter region of androgen receptor gene in 240 men with idiopathic infertility and 30 healthy men and this study did not show presence of any deletions or mutations in infertile or healthy men and they suggested that *AR* gene promoter mutation are not common in men with idiopathic infertility (13). Furthermore a study on *AR* gene promoter region of 100 healthy men suggested that DNA sequence alterations are rare in the human androgen receptor gene promoter (14). 

Additionally, we did not find any of mentioned single nucleotide polymorphisms in control and patient groups using PCR and RFLP techniques. This finding may declare that these five polymorphisms are not be involved in hypospadias etiology. These data are consistent with the previous report by Muroya *et al *in which mutation screening of genomic DNA of men with hypospadias did not show any point mutation in *AR* gene exons (3). Also Hiort *et al* reported that the majority of patients with hypospadias did not carry androgen receptor gene mutations. In addition Sasagawa *et al* suggested that alteration of the *AR* gene is rare in males with isolated hypospadias, cryptorchidism, micropenis, or idiopathic male infertility (5). Furthermore, Radpour *et al* reported that none of the studied hypospadic patients in their research had *AR* gene mutations (15). Likewise, other scientists suggested that *AR* gene mutation is rare in hypospadic patient (16, 17). 

In addition, In a recent study by Adamovic *et al* it was found that a SNP in the AR gene region was associated with susceptibility to severe hypospadias.** They concluded that **AR rs5919436 polymorphism may act as a new gene marker for increased susceptibility to severe hypospadias in Caucasians (18). Besides, another study has investigated the possible association of the CAG repeat length in the *AR* gene with the hypospadias; and suggested that expanded CAG repeat length has a role in modifying the risk and development of hypospadias (19). 

In addition, in another study on Chilean population, the CAG and GGN polymorphisms in the AR gene has been investigated and showed that in isolated hypospadic boys, there are longer CAG alleles in their AR gene that might be associated with the development of hypospadias (20). Taken together, our result suggests that a same nucleotide variation in AR gene promoter of 2 patients with isolated hypospadis may have role in etiology of this congenital abnormality in mentioned patients by reducing the total amount of *AR* gene expression in the genital system due to decreasing the transcription machinery affinity for binding toward regulatory elements.
